# Gender Differences in Symptoms, Health-Related Quality of Life, Sleep Quality, Mental Health, Cognitive Performance, Pain-Cognition, and Positive Health in Spanish Fibromyalgia Individuals: The Al-Ándalus Project

**DOI:** 10.1155/2016/5135176

**Published:** 2016-10-27

**Authors:** Víctor Segura-Jiménez, Fernando Estévez-López, Alberto Soriano-Maldonado, Inmaculada C. Álvarez-Gallardo, Manuel Delgado-Fernández, Jonatan R. Ruiz, Virginia A. Aparicio

**Affiliations:** ^1^Department of Physical Education, Faculty of Education Sciences, University of Cádiz, Cádiz, Spain; ^2^Department of Physical Education and Sport, Faculty of Sport Sciences, University of Granada, Granada, Spain; ^3^Department of Psychology, Faculty of Social and Behavioural Sciences, Utrecht University, Utrecht, Netherlands; ^4^Department of Education, Faculty of Education Sciences, University of Almería, Almería, Spain; ^5^Department of Physiology, Faculty of Pharmacy, University of Granada, Granada, Spain; ^6^Department of Public and Occupational Health, EMGO^+^ Institute for Health and Care Research, VU University Medical Centre, Amsterdam, Netherlands

## Abstract

*Objective*. To test the gender differences in tenderness, impact of fibromyalgia, health-related quality of life, fatigue, sleep quality, mental health, cognitive performance, pain-cognition, and positive health in Spanish fibromyalgia patients and in age-matched nonfibromyalgia individuals from the same region. To test the optimal cut-off score of the different tender points for women and men.* Methods*. A total of 405 (384 women) fibromyalgia versus 247 (195 women) nonfibromyalgia control participants from southern Spain (Andalusia) took part in this cross-sectional study. The outcomes studied were assessed by means of several tests.* Results*. In the fibromyalgia group, men showed better working memory than women (all, *P* < 0.01), whereas sleep latency was lower in women compared to men (*P* = 0.013). In the nonfibromyalgia group, men showed higher pain threshold in all the tender points (all, *P* < 0.01), except in right and left lateral epicondyle. Furthermore, men showed better working memory than women (all, *P* < 0.01), whereas memory performance was better in women compared to men (all, *P* ≤ 0.01).* Conclusion*. The results of the present study do not support consistent evidence of gender differences in fibromyalgia-related symptoms. However, it seems that detriment of some symptoms (especially pain) in fibromyalgia men compared with their nonfibromyalgia counterparts is greater than those of fibromyalgia women compared with their nonfibromyalgia peers.

## 1. Introduction

General prevalence of fibromyalgia varies from 0.5 to 5% depending on the country [1]. The point prevalence of fibromyalgia in Spain is ~2.4%. Remarkably, fibromyalgia is more common in women (~4.2%) than in men (~0.2%) [2]. Due to the low number of diagnosed fibromyalgia men, research work has mainly focused on women, ignoring somewhat the study of fibromyalgia men. Since gender-specific pain mechanisms seem to play a role in general population [3, 4], the idea of gender differences in fibromyalgia symptoms gains veracity. If fibromyalgia women and men present different impact and intensity of their symptoms, diverse and individually tailored diagnosis, management, and treatment would be highly recommended.

The characteristics and symptoms reported by fibromyalgia patients may differ depending on gender. To date, only a few studies have investigated the differential characteristics of fibromyalgia based on gender, showing divergent results and conclusions [5–12], which might be partially explained by sociodemographic and geographical variations among patients [8, 13, 14]. Nonetheless, they could also be related to several methodological weaknesses across previous studies: (i) the majority of previous studies have not controlled their analysis for potential sociodemographic confounders, despite its importance [15]; (ii) several studies did not use standardized validated instruments to assess specific outcomes [8]; (iii) some suggest that studies should compare not only women with chronic pain to men with the same pain condition, but also healthy women to healthy men [16]. None of the previous research has used a group of nonfibromyalgia men and women when studying gender differences in fibromyalgia.

Although previous studies have investigated gender differences in different outcomes in fibromyalgia, sleep is one of the most neglected outcomes until date [17]. Furthermore, gender differences in cognitive performance have not been studied yet, despite its importance as a fibromyalgia symptom [18]. Similarly, a recent term called psychological positive health (hereinafter referred to as positive health), which refers to psychosocial well-being, describes a state beyond the mere absence of disease [19]. The beneficial role of positive health factors on fibromyalgia symptomatology has been reported [20, 21]. For instance, positive affect is inversely associated with fibromyalgia pain and severity [22]. Together with the symptoms traditionally studied, it would be of interest to test gender differences in cognitive performance and positive health in fibromyalgia. We tried to solve the weaknesses of previous studies by (i) introducing a large number of clinical and psychosocial symptoms of the disease assessed with standardized and validated questionnaires, (ii) controlling all the analyses for potential sociodemographic variables, and (iii) including a nonfibromyalgia group of women and men. Therefore, the aim of the present study was to test the gender differences in a pool of relevant symptoms in fibromyalgia such as tenderness, impact of fibromyalgia, health-related quality of life, fatigue, sleep quality, mental health, cognitive performance, pain-cognition, and positive health in Spanish fibromyalgia patients and in age-matched nonfibromyalgia participants from the same demographic area. We also tested the optimal cut-off score of the different tender points for women and men.

## 2. Methods

### 2.1. Participants

Fibromyalgia participants were recruited via fibromyalgia associations from the 8 provinces of Andalusia (Spain) and through e-mail, Internet advertisement, or telephone, which was intended to reach the maximum population of patients with fibromyalgia from this region. We also recruited a group of nonfibromyalgia control participants with similar age, sociodemographic characteristics and demographic area [23]. Interested participants could reach the research team through the fibromyalgia associations or by direct contact through e-mail or telephone. The study assessments were carried out between November 2011 and January 2013. All interested participants (*n* = 960) gave their written informed consent after receiving detailed information about the aims and study procedures. The inclusion criteria for fibromyalgia participants were: (i) to be previously diagnosed of fibromyalgia by a rheumatologist; (ii) to meet the 1990 American College of Rheumatology (ACR) criteria for classification of fibromyalgia [24]; and (iii) not to have acute or terminal illness nor severe cognitive impairment (Mini Mental State Examination (MMSE) < 10) [25]. The inclusion criteria for nonfibromyalgia participants were: (i) not to meet the 1990 ACR fibromyalgia criteria; and (ii) not to have acute or terminal illness nor severe cognitive impairment [25].

The study was reviewed and approved by the Ethics Committee of the* Hospital Virgen de las Nieves* (Granada, Spain).

### 2.2. Procedure

Measurements were performed on two different occasions separated by one day and performed by the same researchers, in order to reduce interexaminers error. On the first day, the MMSE was interviewed and participants filled out self-reported sociodemographic data (age, marital status, educational level, current occupational status, and time since diagnosis) and the Beck Depression Inventory-II (BDI-II). Then, anthropometric measurements and the tender points' examination were assessed. Subsequently, participants received several questionnaires to be filled out at home. At the second appointment, participants returned the questionnaires to the research team and the Paced Auditory Serial Addition Task (PASAT) and the Rey Auditory Verbal Learning Test (RAVLT) were interviewed.

### 2.3. Outcome Measures

#### 2.3.1. Body Mass Index

We measured weight with a bioelectrical impedanciometer (InBody R20; Biospace, Seoul, Korea) and height (cm) using a stadiometer (Seca 22, Hamburg, Germany). Body mass index was calculated as weight (kg) divided by the square of the height (m).

#### 2.3.2. Tenderness

*Tender Points' Examination*. We assessed 18 tender points according to the 1990 ACR criteria for classification of fibromyalgia [24] using a standard pressure algometer (FPK 20; Wagner Instruments, Greenwich, CT, USA). A tender point was scored as positive when patient felt pain at a mechanical pressure ≤ 4 kg/cm^2^. The total count of positive tender points (tender points count) was recorded for each participant. Patients were considered to have fibromyalgia if they had 11 or more positive tender points. Total pain threshold was calculated as the sum of the minimum pain-pressure values obtained for each tender point. One trained researcher performed all the tender points' examinations.

#### 2.3.3. Impact of Fibromyalgia

*The Revised Fibromyalgia Impact Questionnaire (FIQR)* comprises 21 individual questions with a rating scale of 0 to 10. These questions compose 3 different domains: function, overall impact, and symptoms score (ranging 0–30, 0–20, and 0–50, resp.) [26, 27]. The FIQR total score ranges from 0 to 100, with a higher score indicating greater impact. The Symptom Impact Questionnaire (a slightly modified version of the FIQR to be used with healthy individuals) was used with nonfibromyalgia participants [28].

#### 2.3.4. Health-Related Quality of Life

The 36-item* Short-Form Health Survey 36* is a generic instrument for assessing health-related quality of life [29, 30]. It contains 36 items grouped into 8 dimensions: physical functioning, physical role, body pain, general health, vitality, social functioning, emotional role, and mental health. The score ranges from 0 to 100 in every dimension, where higher score indicates better health. The standardized physical component (range 0–100) and the standardized mental component (range 0–100) were also calculated.

#### 2.3.5. Fatigue

*The Multidimensional Fatigue Inventory (MFI)* was used to measure fatigue severity. Five subscales compose this questionnaire: general fatigue, physical fatigue, mental fatigue, reduced activity, and reduced motivation [31, 32]. Each subscale includes four items with 5-point Likert scales. Score on each subscale ranges from 4 to 20, with higher score indicating greater fatigue.

#### 2.3.6. Sleep Quality

*The Pittsburgh Sleep Quality Index (PSQI) questionnaire* was used to assess sleep quality and disturbances over the last month [33, 34]. Nineteen individual items generate seven component scores: subjective sleep quality, sleep latency, sleep duration, habitual sleep efficiency, sleep disturbances, use of sleeping medication, and daytime dysfunction. The sum of scores for these seven components yields one global score (0–21), with higher score indicating worse sleep quality.

#### 2.3.7. Mental Health

*The MMSE* is a brief cognitive screening test used to evaluate cognitive capacity and severity of cognitive impairment [35, 36]. It contains 30 items and the range of score is 0–30, with lower score indicating greater cognitive impairment.

*The BDI-II* was used to assess depression severity [37, 38]. It contains 21 items and the range of score is 0–63 with higher values indicating greater depression.

The* State Trait Anxiety Inventory-I* was used to assess anxiety state (i.e., the level of current anxiety) [39, 40]. It is a 20-item self-administered questionnaire and the range of score is 20–80, with higher score indicating a greater anxiety state.

#### 2.3.8. Pain-Cognition

*The chronic pain self-efficacy scale* was used to assess participants' believed ability to achieve specific outcomes for coping with pain [41, 42]. The 19 items are grouped into 3 subscales (ranging 0–100): pain management, coping with symptoms, and physical function. The total score is the sum of the three subscales (ranging 0–300), where higher score indicates higher self-efficacy.

*The Pain Catastrophizing Scale* [43, 44] was used to assess painful experiences and thoughts or feelings about pain. It contains 13 items on a 5-point scale. For this study, the total score (ranging from 0 to 52) was used, where higher score represents a more negative appraisal of pain.

#### 2.3.9. Cognitive Performance

*The PASAT* [45] was used to measure working memory. It was administered at the slowest presentation rate of 2.4 seconds. The score is the number of correct responses over 60 trials.

*The RAVLT* [46, 47] is a multiple-trial verbal list learning test. In the first trial (A1) the interviewer pronounces aloud a list of 15 words. After finishing, the participant has to repeat all the words remembered. The same procedure is followed across 4 trials (A2, A3, A4, and A5) with the same words. Subsequently a list of 15 different words is presented (B1). Finally, the participant has to remember as many words as possible from the first list (A6). After 20 minutes of the last trial, the participant is asked again to remember as many words as possible from the first list (A7). Lastly, the interviewer exposes a list of 50 words (recognition matrix) and the participant has to remember if they belonged to trial A, trial B, or none of them. The correct answers compose the score of each trial and the recognition matrix. This questionnaire measures immediate free recall, delayed free recall, verbal learning, and delayed recognition.

#### 2.3.10. Positive Health

*The Life Orientation Test Revised* [48, 49] assesses participants' expectations about their future and their general sense of optimism. It contains 10 items rated on a 5-point Likert scale. The higher the score obtained in the test, the higher the level of dispositional optimism.

*The Positive and Negative Affect Schedule* [50–52] is a 20-item questionnaire designed to measure the emotional component of subjective well-being. Its items group into two subscales: positive and negative affect. Higher score indicates higher positive affect or negative affect.

*The Satisfaction with Life Scale* [53, 54] assesses the cognitive component of subjective well-being. It consists of five items with a 7-point Likert scale. Higher score indicates greater satisfaction with life.

*The Trait Metamood Scale* [55, 56] has three subscales. In the present study we only used the mood repair subscale (8 items), which assesses how well individuals regulate their moods and repair negative emotional experiences. Responses are rated using a 5-point Likert scale. The final score goes from 8 up to 40, with higher values indicating better mood repair.

### 2.4. Statistical Analysis

Analyses were performed in fibromyalgia patients and nonfibromyalgia participants separately. Since variables were nonnormally distributed, the Mann-Whitney test was used to analyze the differences in continuous sociodemographic variables between women and men groups. The Chi-square test was used for sociodemographic categorical variables. To test the gender differences in symptoms, linear regression was performed for each dependent variable (study outcomes). The independent variable was gender (woman versus man). Marital status and current occupational status (plus body mass index in the nonfibromyalgia group) were used as covariates in all the analyses, since they were statistically different between men and women. The presence of interaction was also studied. For that purpose, all participants were included in the same analysis with condition (fibromyalgia versus nonfibromyalgia), gender (woman versus man), and the interaction term (condition × gender) as independent variables, while controlling for the covariates previously reported. For better clarity and representation of the results, data were presented as mean (standard error). Cohen's *d* was used to calculate the effect size of the differences [57]. To corroborate that tenderness presents gender-specific mechanisms in fibromyalgia, the optimal cut-off score of the different tender points was studied by using the receiving operator characteristics in women and men separately. Due to the exploratory nature of this analysis and to avoid the influence of the tender points' criteria selection, the rheumatologist criterion was selected as the case definition in this particular case. This approach has been previously used in the literature to assess the validity of diagnostic criteria in fibromyalgia [24, 58, 59]. The Statistical Package for Social Sciences (IBM SPSS Statistics for Windows, version 20.0, Armonk, NY, USA) was used. Due to the multiple comparisons, the level of significance was set at *P* ≤ 0.01 (two-tailed).

## 3. Results

**Figure 1:**
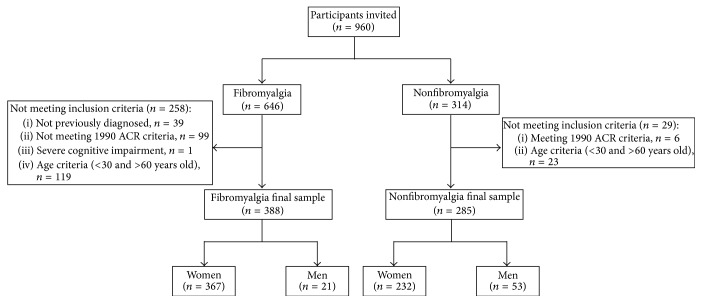
Flow diagram of participants.

Thirty-nine* a priori* fibromyalgia patients were not previously diagnosed; 99* a priori* fibromyalgia patients did not meet the 1990 ACR criteria whereas 6* a priori* nonfibromyalgia individuals met the criteria. Additionally 1 fibromyalgia patient had severe cognitive impairment assessed by MMSE. In order to obtain age-matched groups, participants <30 and >60 years old were excluded. One hundred and forty-two individuals did not meet the age criteria. The final study sample comprised 388 fibromyalgia (367 women, 21 men) versus 285 nonfibromyalgia (232 women, 53 men) participants from southern Spain (Andalusia). The flow diagram of participants is displayed in Figure 1.

The sociodemographic variables of the study groups are shown in Table 1. In both fibromyalgia and nonfibromyalgia groups, there were significant gender differences in marital status (*P* < 0.01), with a lower percentage of single women than single men. In both fibromyalgia and nonfibromyalgia groups, the occupational status differed between genders (*P* < 0.001) with greater housewife and lower not working percentages of women than men.

Tender points of the study participants by groups are shown in Table 2. In the fibromyalgia group there were no differences in tenderness between women and men (all, *P* > 0.05). In the nonfibromyalgia group, tenderness differed between women and men, where women displayed lower values (*P* < 0.01), except for the left and right epicondyle (*P* > 0.05). Consequently, the total number of tender points was higher and the total pain threshold was lower in women compared to men (3.3 versus 0.8 kg/cm^2^, *P* < 0.001, and 105.9 versus 127.6 kg/cm^2^, *P* < 0.001, resp.). In addition, we found a statistically significant interaction in all the variables assessing tenderness, except in the right and left lateral epicondyle and gluteus (all, *P* ≤ 0.002).

The impact of fibromyalgia, health-related quality of life, fatigue, and sleep quality of the fibromyalgia and nonfibromyalgia participants are shown in Table 3. In the fibromyalgia group, women showed borderline significant lower sleep latency (47.9 versus 72.7 minutes, *P* = 0.013) than men. In both fibromyalgia and nonfibromyalgia groups, women showed a nonsignificant trend towards presenting lower values in reduced activity from the MFI (*P* ≤ 0.038). We found a statistically significant interaction in sleep latency (*P* = 0.011). No differences between groups were observed regarding the rest of outcomes.

Mental health, pain-cognition, and cognitive performance of the study groups are presented in Table 4. In the fibromyalgia group, there were no differences in any of the variables studied except for the PASAT, with women showing lower number of “correct answers” (32.4 versus 40.0; *P* < 0.01) and higher number of “not answered” questions (20.1 versus 11.8; *P* < 0.01) than men. In the nonfibromyalgia group, women showed higher values of depression (10.4 versus 6.5; *P* < 0.01) and borderline significant higher anxiety (20.4 versus 15.8; *P* = 0.013) than men. We found a borderline significant interaction in depression (*P* = 0.053) and a statistically significant interaction in anxiety (*P* = 0.009). Nonfibromyalgia women showed lower number of “correct answers” (35.1 versus 40.5; *P* = 0.01) and nonsignificant trend towards higher number of “not answered” questions (17.1 versus 12.7; *P* = 0.016) than nonfibromyalgia men. Nonfibromyalgia women showed borderline significant higher immediate memory (65.8 versus 60.9; *P* = 0.015), delay recall (10.5 versus 9.4; *P* = 0.016), and statistically significant higher verbal learning (49.8 versus 45.8; *P* < 0.01) than nonfibromyalgia men.

Table 5 shows the positive health of the study groups. In both fibromyalgia and nonfibromyalgia groups, there were no gender differences in any of the variables studied (all, *P* > 0.05). A borderline significant interaction was found in negative affect (*P* = 0.049).

The study of sensitivity and specificity of the different tender points showed that women and men present different optimal cut-offs for the fibromyalgia diagnosis (all, *P* < 0.001), with men showing higher pain thresholds than women (Table 6).

## 4. Discussion

We aimed at studying the gender differences in a large variety of symptoms assessed with standardized and validated questionnaires in fibromyalgia as well as in nonfibromyalgia participants. From all the variables studied, fibromyalgia women showed better sleep latency and lower working memory than men did. In the nonfibromyalgia group, women showed higher pain sensitivity, worse mental health status, and lower working memory, whereas they presented better memory than men did.

In the general population, women usually present greater pain sensitivity and lower pain threshold than men [60], which is in agreement with the results found in the nonfibromyalgia group of the present study. It has been speculated that both peripheral and central nervous systems pathways might be involved in pain experiences; however, the mechanism underlying gender differences in pain remains misunderstood [61]. Previous research in fibromyalgia has shown different results: some of them presenting more pain or tenderness in women than men [7, 8, 11, 62], whereas others display no gender differences [15, 17]. The difference between these previous studies and ours might rely on the fact that they did not control for key sociodemographic variables, which could have altered the results. Furthermore, cross-cultural differences might also be involved in the different results observed in the current literature [8]. Although no gender differences were observed in pain in the fibromyalgia group, the nonfibromyalgia women from the present study showed higher pain sensitivity and lower pain thresholds than nonfibromyalgia men, with large effect sizes. This might suggest that fibromyalgia impacts men more severely than women regarding pain tolerance, which invites one to think that the tender points' diagnostic criteria should be gender-tailored, as corroborated with the ROC analyses in the present study. According to our results, the cut-off for fibromyalgia diagnosis in men should be greater than those of the women. In fact, the average of gender-based optimal cut-off in the present study was 3.8 kg/cm^2^ for women whereas it was 5.1 kg/cm^2^ for men. If we weight the average values above for women and men based on their respective contributions to the total sample ([3.8 kg/cm^2^  ×  0.88] + [5.1 kg/cm^2^  ×  0.12]), the composite value results in 3.95 kg/cm^2^, which concurs with the 4.0 kg/cm^2^ cut-off settled upon in the 1990 ACR criteria. However, we have shown that the use of a 4.0 kg/cm^2^ cut-off of pressure for fibromyalgia diagnosis purpose presents a handicap for men, and this might be one of the reasons why fewer men are usually diagnosed. Furthermore, we showed that diverse anatomical locations present different pressure pain sensitivity and, as a consequence, different cut-offs should be used for diagnostic criteria purpose [63, 64]. Our results, then, suggest that fibromyalgia pain might be aggravated in men and, consequently, there might be gender-specific pain mechanisms in fibromyalgia, which concurs with the findings suggesting gender differences in neural responses to pain [65]. Nonetheless, given the low sample size of our sample, our findings should be interpreted as preliminary and future studies with a larger sample size of men might confirm or contrast the cut-off scores suggested in the present study.

Previous research has shown that fibromyalgia men present more severe limitations in physical functioning [8, 11, 12], social functioning, and health perception [9]. However, we failed to find these differences between fibromyalgia women and men in the present study. Our results are consistent with other studies finding no gender differences in clinical key features in fibromyalgia [15, 17, 62]. Regarding fatigue, it is noteworthy that, in general population, women usually present greater values than men [66]. By contrast, we did not find significant differences in all the fatigue dimensions from the MFI, except a trend of women reporting less fatigue than men in the reduced activity dimension in both the fibromyalgia and nonfibromyalgia groups. The explanation of these results is uncertain, and further research is warranted.

Sleep quality, assessed with a VAS scale, has been inversely related to quality of life in fibromyalgia men but not in women [8]. This may suggest that sleep disturbances in fibromyalgia might affect men more severely than women. Another study in a Spanish population reported no gender differences in the global scale of the PSQI in fibromyalgia [62], which concurs with our results. However, we found that fibromyalgia men reported greater sleep latency and a trend towards worse sleep efficiency compared to women. These findings are in agreement with a recent polysomnographic study where fibromyalgia men showed more sleep complaints than women [17].

Yunus et al. [6] were the first studying the psychological status of male patients with fibromyalgia compared with females. They concluded that nonnotable gender differences were encountered. The same conclusion was reached in another study which tested psychological differences between fibromyalgia women and men [15, 62]. In the present study, although the levels of depression and anxiety observed in fibromyalgia men are comparable to those observed in fibromyalgia women, the lower values observed in nonfibromyalgia men compared to nonfibromyalgia women and the presence of a statistically significant interaction term in the regression analysis invite us to think that fibromyalgia might affect mental health more severely in men than women. An explanation might be that fibromyalgia is more prevalent in women than in men [2, 67, 68] and is popularly understood as a “female disease.” Furthermore, men might assume worse than women those limitations and impairments imposed by fibromyalgia. Therefore, gender-specific psychological factors [69] might be present in fibromyalgia.

In the current study, both fibromyalgia and nonfibromyalgia men presented a better working memory than women when performing the PASAT. Furthermore, it is noteworthy to highlight that all participants in both fibromyalgia and nonfibromyalgia groups showed similar mean values. Contrary to the body of literature [18, 70–72], this fact suggests that computing issues are not importantly affected among Spanish patients with fibromyalgia syndrome. Likewise, the scoring with the RAVLT was very similar in fibromyalgia and nonfibromyalgia, and no gender differences were observed. According to these results, fibromyalgia patients might not be so severely affected by memory and cognitive problems as it is usually reported [18, 70]. Although this observation might be shocking, we must bear in mind that the majority of studies investigating cognitive function in fibromyalgia have used self-report measurements [72]. It has been stated that fibromyalgia patients could possibly overstate their memory deficits when assessed by means of self-report measurements [73].

Pain-cognition severity, such as pain self-efficacy and catastrophizing, might differ depending on gender in the general population [3]. However, we did not find gender differences in the present study. Our results are consistent with a previous study testing the gender differences in the Pain Catastrophizing Scale in Spanish fibromyalgia patients [62]. Although no gender differences in pain-cognition profile in fibromyalgia were observed, it is noteworthy that pain perception is mainly related to cognitive-affective factors, especially in women [62]. Similarly, although fibromyalgia seems to impact patients' positive health [21], the absence of gender differences in the present study suggests that this condition equally impairs the positive health of fibromyalgia men and women.

Some limitations must be mentioned. First, the cross-sectional design does not allow establishing causal relationships. Second, the possibility of type I error, even with multiple testing correction, is a limitation of the study. Third, the low sample size of men might have masked some statistically significant analyses and diminish the accuracy of the results, due to the lack of statistical power. Therefore, the results observed in the present study must be interpreted cautiously. Nonetheless, the lower prevalence of fibromyalgia in men and their low rate of participation in research studies have led to overall low sample sizes of men in studies performed to date [6, 8, 9, 11, 12, 17, 62]. Furthermore, according to the most recent study of the prevalence of fibromyalgia in Spain [2], the women/men fibromyalgia proportion is approximately 22 : 1, which approximately fits with the ratio of women and men recruited in our study (17.5 : 1). A strength of the present study was the use of standardized and validated tests to assess the diverse symptoms. The large number of outcomes studied provides a general knowledge about gender differences in many fibromyalgia-related symptoms. Furthermore, the adjustment for potential sociodemographic variables in the statistical analyses was another strength of the study. Finally, the inclusion of a nonfibromyalgia group allowed us to obtain novel conclusions, which could have been masked without the participation of this group.

## 5. Conclusions

The findings of the present study did not support consistent gender-specific differences in fibromyalgia symptoms. Nonetheless, the results seem to show that fibromyalgia might affect more severely men than women regarding tenderness, mental health, and sleep latency. Therefore, when research is focused on the aforementioned symptoms, it might be advisable to study women and men separately. Nevertheless, future studies with larger men sample size should confirm or contrast the present results.

## Figures and Tables

**Table 1:** Clinical and sociodemographic variables in women and men, separated by the presence or absence of fibromyalgia.

	Fibromyalgia		*P*_gender_	Nonfibromyalgia		*P*_gender_
	Women (*n* = 367)	Men (*n* = 21)		Women (*n* = 232)	Men (*n* = 53)	
	Mean (SD)	Mean (SD)		Mean (SD)	Mean (SD)	
Age, years	49.0 (6.0)	46.9 (8.4)	0.074	50.0 (8.1)	48.1 (10.6)	0.332
Body mass index, kg/cm^2^	28.2 (5.8)	28.1 (4.8)	0.880	26.6 (4.3)	28.5 (3.8)	0.001

	*n* (%)	*n* (%)		*n* (%)	*n* (%)	

Marital status			0.009			0.003
Married	279 (76.0)	13 (61.9)		170 (73.6)	38 (71.7)	
Single	33 (9.0)	7 (33.3)		23 (10.0)	14 (26.4)	
Separated	10 (2.7)	0 (0.0)		13 (5.6)	0 (0.0)	
Divorced	31 (8.4)	1 (4.8)		15 (6.5)	1 (1.9)	
Widow(er)	14 (3.8)	0 (0.0)		10 (4.3)	0 (0.0)	
Educational level			0.855			0.281
No studies/primary school	196 (53.4)	10 (47.7)		103 (44.4)	21 (39.7)	
Secondary school	114 (31.1)	7 (33.3)		75 (32.3)	23 (43.4)	
University degree	57 (15.5)	4 (19.0)		54 (23.2)	9 (16.9)	
Current occupational status			<0.001			<0.001
Working	109 (29.7)	3 (14.3)		95 (40.9)	31 (58.5)	
Housewife	102 (27.8)	0 (0.0)		82 (35.3)	0 (0.0)	
Not working	156 (42.5)	18 (85.7)		55 (23.7)	22 (41.5)	
Time since diagnosis			0.640			
Less than 5 years	173 (47.1)	11 (52.4)				
More than 5 years	194 (55.8)	10 (50.0)				

SD, standard deviation.

**Table 2:** Tender points in women and men, separated by the presence or absence of fibromyalgia.

	Fibromyalgia		*P*	Effect size	Nonfibromyalgia		*P*	Effect size	*P*_interaction_
	Women (*n* = 367)	Men (*n* = 21)			Women (*n* = 231)	Men (*n* = 52)			
	Mean (SE)	Mean (SE)			Mean (SE)	Mean (SE)			
Occiput right total	2.2 (0.0)	2.3 (0.2)	0.610	0.11	5.5 (0.1)	6.6 (0.2)	<0.001	0.65	0.008
Occiput left total	2.2 (0.0)	2.3 (0.2)	0.665	0.10	5.5 (0.1)	6.5 (0.2)	<0.001	0.64	0.008
Anterior cervical right total	1.7 (0.0)	1.7 (0.1)	0.991	0.00	4.2 (0.1)	5.6 (0.2)	<0.001	0.84	<0.001
Anterior cervical left total	1.7 (0.0)	1.7 (0.1)	0.742	0.07	4.0 (0.1)	5.4 (0.2)	<0.001	0.92	<0.001
Trapezius right total	2.3 (0.0)	2.4 (0.2)	0.964	0.01	6.1 (0.1)	7.3 (0.2)	<0.001	0.83	<0.001
Trapezius left total	2.4 (0.0)	2.3 (0.2)	0.408	0.18	6.1 (0.1)	7.3 (0.2)	<0.001	0.86	<0.001
Supraspinatus right total	2.6 (0.1)	2.2 (0.2)	0.100	0.37	6.8 (0.1)	7.6 (0.2)	<0.001	0.62	<0.001
Supraspinatus left total	2.7 (0.1)	2.4 (0.2)	0.182	0.30	7.1 (0.1)	7.7 (0.2)	<0.001	0.57	0.003
Second rib right total	2.1 (0.0)	2.2 (0.2)	0.453	0.17	5.2 (0.1)	7.1 (0.2)	<0.001	1.22	<0.001
Second rib left total	2.0 (0.0)	2.2 (0.2)	0.154	0.32	5.0 (0.1)	7.2 (0.2)	<0.001	1.35	<0.001
Lateral epicondyle right total	3.0 (0.1)	2.6 (0.3)	0.246	0.26	7.2 (0.1)	7.3 (0.2)	0.503	0.10	0.178
Lateral epicondyle left total	2.9 (0.1)	2.5 (0.3)	0.213	0.28	7.0 (0.1)	7.3 (0.2)	0.207	0.20	0.085
Gluteal right total	2.5 (0.1)	2.9 (0.2)	0.157	0.31	6.5 (0.1)	7.7 (0.2)	<0.001	0.83	0.020
Gluteal left total	2.6 (0.1)	3.0 (0.2)	0.047	0.44	6.3 (0.1)	7.6 (0.2)	<0.001	0.89	0.023
Great trochanter right total	2.7 (0.1)	2.8 (0.2)	0.494	0.15	6.5 (0.1)	7.7 (0.2)	<0.001	0.85	0.005
Great trochanter left total	2.6 (0.0)	2.8 (0.2)	0.224	0.27	6.2 (0.1)	7.6 (0.2)	<0.001	0.93	0.002
Knee right total	2.1 (0.0)	2.4 (0.2)	0.089	0.38	5.4 (0.1)	7.0 (0.2)	<0.001	0.92	<0.001
Knee left total	2.1 (0.0)	2.4 (0.2)	0.128	0.34	5.4 (0.1)	7.2 (0.2)	<0.001	1.03	<0.001
Total number points	16.8 (0.1)	16.8 (0.4)	0.877	0.04	3.3 (0.2)	0.8 (0.4)	<0.001	0.88	<0.001
Total pain threshold	42.6 (0.7)	43.2 (3.0)	0.829	0.05	105.9 (1.4)	127.6 (3.0)	<0.001	1.03	<0.001

SE, standard error. Differences between women and men groups were performed using linear regression with marital status and current occupational status entered as covariates in fibromyalgia, whereas marital status, current occupational status, and body mass index were used in nonfibromyalgia participants. Effect size statistics are expressed as Cohen's *d*.

**Table 3:** Fibromyalgia impact, health-related quality of life, fatigue, and sleep in women and men by presence or absence of fibromyalgia.

	Fibromyalgia			*P*	Effect size	Nonfibromyalgia		*P*	Effect size	*P*_interaction_
	Women (*n* = 354)	Men (*n* = 21)				Women (*n* = 217)	Men (*n* = 49)			
	Mean (SE)	Mean (SE)				Mean (SE)	Mean (SE)			
FIQR										
Function (0–30)	17.2 (0.3)	16.0 (1.4)		0.380	0.20	2.4 (0.3)	1.3 (0.5)	0.073	0.29	0.926
Overall impact (0–20)	12.7 (0.3)	14.1 (1.1)		0.250	0.26	2.4 (0.3)	2.3 (0.6)	0.932	0.01	0.234
Symptoms score (0–50)	34.8 (0.4)	35.5 (1.7)		0.700	0.09	15.9 (0.5)	15.0 (1.2)	0.503	0.11	0.430
Total score (0–100)	64.7 (0.9)	65.5 (3.6)		0.837	0.05	20.7 (0.9)	18.7 (1.9)	0.339	0.15	0.449
SF-36										
Physical component (0–100)	29.5 (0.4)	30.4 (1.5)		0.516	0.15	47.9 (0.6)	50.5 (1.3)	0.065	0.29	0.507
Mental component (0–100)	35.7 (0.6)	33.0 (2.6)		0.325	0.22	48.2 (0.8)	51.4 (1.6)	0.083	0.28	0.068
MFI										
General fatigue (4–20)	18.2 (0.1)	18.5 (0.5)		0.523	0.15	10.5 (0.3)	9.4 (0.6)	0.122	0.25	0.166
Physical fatigue (4–20)	16.9 (0.2)	16.7 (0.6)		0.758	0.07	10.2 (0.3)	9.7 (0.6)	0.456	0.12	0.717
Reduced activity (4–20)	13.1 (0.3)	15.3 (1.1)		0.038	0.47	7.6 (0.3)	9.0 (0.6)	0.030	0.35	0.390
Reduced motivation (4–20)	13.3 (0.2)	13.9 (0.8)		0.461	0.17	8.5 (0.2)	7.5 (0.5)	0.072	0.29	0.098
Mental fatigue (4–20)	14.4 (0.1)	14.6 (0.5)		0.625	0.11	11.5 (0.2)	11.7 (0.4)	0.662	0.07	0.939
PSQI										
Sleep duration (min/day)	342.1 (4.6)	321.4 (19.1)		0.292	0.24	410.4 (4.8)	410.7 (10.1)	0.979	0.00	0.403
Sleep latency (min/day)	47.9 (2.3)	72.7 (9.7)		0.013	0.57	22.8 (1.8)	21.6 (3.7)	0.769	0.05	0.011
Sleep efficiency (%)	71.1 (1.0)	64.0 (4.0)		0.080	0.40	86.5 (0.9)	87.4 (1.9)	0.666	0.07	0.072
Global score (0–21)	13.3 (0.2)	14.3 (0.8)		0.238	0.27	6.5 (0.3)	6.0 (0.6)	0.403	0.13	0.187

FIQR, Revised Fibromyalgia Impact Questionnaire. MFI, Multidimensional Fatigue Inventory. PSQI, Pittsburgh Sleep Quality Index. SD, standard deviation. SF-36, 36-item Short-Form Health Survey. Differences between women and men groups were performed using linear regression with marital status and current occupational status entered as covariates in fibromyalgia, whereas marital status, current occupational status, and body mass index were used in nonfibromyalgia participants. Effect size statistics are expressed as Cohen's *d*.

**Table 4:** Mental health, pain-cognition, and cognitive performance in women and men, separated by the presence or absence of fibromyalgia.

	Fibromyalgia		*P*	Effect size	Nonfibromyalgia		*P*	Effect size	*P*_interaction_
	Women (*n* = 367)	Men (*n* = 21)			Women (*n* = 231)	Men (*n* = 52)			
	Mean (SE)	Mean (SE)			Mean (SE)	Mean (SE)			
MMSE (0–30)	28.1 (0.1)	28.2 (0.5)	0.948	0.01	28.4 (0.1)	28.6 (0.3)	0.499	0.11	0.843
BDI-II (0–63)	26.7 (0.6)	28.4 (2.6)	0.525	0.14	10.4 (0.6)	6.5 (1.2)	0.004	0.46	0.053
STAI (20–80)	35.4 (0.6)	39.7 (2.7)	0.120	0.35	20.4 (0.8)	15.8 (1.7)	0.013	0.39	0.009
Chronic pain self-efficacy									
Copying (0–100)	48.1 (1.1)	42.5 (4.6)	0.238	0.26	72.5 (1.4)	73.5 (3.1)	0.765	0.05	0.177
Function (0–100)	54.5 (1.2)	60.4 (4.8)	0.232	0.27	86.7 (1.4)	88.6 (3.0)	0.572	0.09	0.522
Pain (0–100)	34.0 (1.2)	33.3 (4.9)	0.889	0.03	68.6 (1.7)	66.3 (3.7)	0.575	0.09	0.796
Total score (0–300)	136.6 (2.9)	136.2 (12.0)	0.973	0.01	227.8 (3.8)	228.4 (8.0)	0.944	0.01	0.878
Catastrophizing									
Rumination (0–16)	8.6 (0.2)	9.0 (1.0)	0.708	0.08	4.4 (0.3)	4.2 (0.6)	0.813	0.04	0.536
Magnification (0–12)	5.0 (0.2)	5.1 (0.7)	0.885	0.03	2.3 (0.2)	1.9 (0.4)	0.347	0.15	0.391
Hopelessness (0–24)	11.5 (0.3)	12.1 (1.3)	0.677	0.09	4.5 (0.3)	3.7 (0.7)	0.293	0.16	0.302
Total score (0–52)	25.1 (0.7)	26.2 (2.7)	0.712	0.08	11.2 (0.7)	9.9 (1.5)	0.427	0.12	0.353
PASAT									
Correct answers (0–60)	32.4 (0.6)	40.0 (2.7)	0.005	0.63	35.1 (0.9)	40.5 (1.9)	0.010	0.40	0.459
Wrong answers (0–60)	7.5 (0.3)	8.2 (1.3)	0.602	0.12	7.8 (0.4)	6.8 (0.8)	0.232	0.19	0.357
Not answered (0–60)	20.1 (0.6)	11.8 (2.4)	0.001	0.77	17.1 (0.8)	12.7 (1.6)	0.016	0.37	0.187
RAVLT									
Immediate memory (0–105)	63.0 (0.6)	59.3 (2.7)	0.193	0.29	65.8 (0.9)	60.9 (1.8)	0.015	0.38	0.569
Delayed recall (0–15)	9.6 (0.2)	8.8 (0.7)	0.260	0.25	10.5 (0.2)	9.4 (0.4)	0.016	0.37	0.574
Verbal learning (0–75)	47.7 (0.5)	44.9 (2.0)	0.185	0.30	49.8 (0.6)	45.8 (1.3)	0.006	0.43	0.512
Recognition memory (0–50)	35.5 (0.3)	34.5 (1.2)	0.432	0.18	36.3 (0.4)	35.5 (0.8)	0.366	0.14	0.884

BDI-II, Beck Depression Inventory-II. MMSE, Mini Mental State Examination. PASAT, Paced Auditory Serial Addition Task. RAVLT, Rey Auditory Verbal Learning Test. SD, standard deviation. Differences between women and men groups were performed using linear regression with marital status and current occupational status entered as covariates in fibromyalgia, whereas marital status, current occupational status, and body mass index were used in nonfibromyalgia participants. Effect size statistics are expressed as Cohen's *d*.

**Table 5:** Psychological positive health in women and men, separated by the presence or absence of fibromyalgia.

	Fibromyalgia		*P*	Effect size	Nonfibromyalgia		*P*	Effect size	*P*_interaction_
	Women (*n* = 354)	Men (*n* = 21)			Women (*n* = 219)	Men (*n* = 50)			
	Mean (SE)	Mean (SE)			Mean (SE)	Mean (SE)			
LOTR									
Optimism (0–12)	6.9 (0.1)	6.2 (0.6)	0.240	0.27	8.3 (0.2)	8.5 (0.4)	0.768	0.05	0.186
Pessimism (0–12)	6.6 (0.1)	5.8 (0.6)	0.199	0.29	7.5 (0.2)	7.4 (0.4)	0.770	0.05	0.404
PANAS									
Positive affect (10–50)	22.7 (0.4)	21.8 (1.5)	0.560	0.13	30.9 (0.6)	30.3 (1.2)	0.648	0.07	0.898
Negative affect (10–50)	24.6 (0.5)	27.5 (1.9)	0.139	0.34	17.8 (0.4)	16.3 (0.9)	0.131	0.24	0.049
SWLS									
Satisfaction with life (5–25)	14.0 (0.2)	13.1 (1.0)	0.402	0.19	17.9 (0.3)	17.6 (0.6)	0.615	0.08	0.536
TMMS									
Emotional repair (8–40)	22.9 (0.4)	20.3 (1.6)	0.130	0.34	26.9 (0.5)	27.0 (1.1)	0.954	0.01	0.206

LOT-R, Life Orientation Test Revised. PANAS, Positive and Negative Affect Schedule. SE, standard error. SWLS, Satisfaction with Life Scale. TMMS, Trait Metamood Scale. Differences between women and men groups were performed using linear regression with marital status and current occupational status entered as covariates in fibromyalgia, whereas marital status, current occupational status, and body mass index were used in nonfibromyalgia participants. Effect size statistics are expressed as Cohen's *d*.

**Table 6:** Test characteristics of tender points' criteria for classifying fibromyalgia using clinical diagnosis as the gold standard in both fibromyalgia and nonfibromyalgia women (*n* = 666) and men (*n* = 82).

Tender points location	Women				Men			
	Optimal cut-off	AUC	Sensitivity	Specificity	Optimal cut-off	AUC	Sensitivity	Specificity
Occiput	**3.7**	0.93	0.86	0.89	**4.2**	0.96	0.89	0.93
Anterior cervical	**2.4**	0.92	0.80	0.92	**2.9**	0.97	0.96	0.91
Trapezius	**4.3**	0.94	0.87	0.91	**4.7**	0.99	0.93	0.94
Supraspinatus	**4.6**	0.95	0.82	0.95	**6.2**	0.99	0.96	0.94
Second rib	**3.3**	0.91	0.82	0.85	**5.0**	0.98	1.00	0.89
Lateral epicondyle	**4.8**	0.92	0.78	0.94	**5.6**	0.97	0.96	0.87
Gluteal	**4.1**	0.93	0.81	0.93	**6.5**	0.97	1.00	0.89
Great trochanter	**3.9**	0.93	0.79	0.97	**5.6**	0.96	0.89	0.93
Knee	**3.2**	0.93	0.81	0.91	**5.0**	0.95	0.93	0.83

The optimal cut-off was selected using the best balanced accuracy ([sensitivity + specificity]/2) for the different possible cut-offs. Each tender point consists of the mean of left and right tender body sides (e.g., occiput = (occiput right + occiput left)/2). AUC, area under the curve. All *P* < 0.001.
